# Soft CNT-Polymer Composites for High Pressure Sensors

**DOI:** 10.3390/s22145268

**Published:** 2022-07-14

**Authors:** Adebayo Eisape, Valerie Rennoll, Tessa Van Volkenburg, Zhiyong Xia, James E. West, Sung Hoon Kang

**Affiliations:** 1 Department of Electrical and Computer Engineering, Johns Hopkins University, Baltimore, MD 21218, USA; aeisape1@jhu.edu (A.E.); vrennol1@jhu.edu (V.R.); jimwest@jhu.edu (J.E.W.); 2 Research and Exploratory Development Department (REDD), Johns Hopkins University Applied Physics Laboratory, Laurel, MD 20723, USA; tessa.vanvolkenburg@jhuapl.edu (T.V.V.); zhiyong.xia@jhuapl.edu (Z.X.); 3 Department of Mechanical Engineering, Hopkins Extreme Materials Institute (HEMI), Johns Hopkins University, Baltimore, MD 21218, USA

**Keywords:** composite, thermoplastic polyurethane (TPU), multiwalled carbon nanotube (MWCNT), piezoresistive, high pressure sensor

## Abstract

Carbon–polymer composite-based pressure sensors have many attractive features, including low cost, easy integration, and facile fabrication. Previous studies on carbon–polymer composite sensors focused on very high sensitivities for low pressure ranges (10 s of kPa), which saturate quickly at higher pressures and thus are ill-suited to measure the high pressure ranges found in various applications, including those in underwater (>1 atm, 101 kPa) and industrial environments. Current sensors designed for high pressure environments are often difficult to fabricate, expensive, and, similarly to their low-pressure counterparts, have a narrow sensing range. To address these issues, this work reports the design, synthesis, characterization, and analysis of high-pressure TPU-MWCNT based composite sensors, which detect pressures from 0.5 MPa (4.9 atm) to over 10 MPa (98.7 atm). In this study, the typical approach to improve sensitivity by increasing conductive additive concentration was found to decrease sensor performance at elevated pressures. It is shown that a better approach to elevated pressure sensitivity is to increase sensor response range by decreasing the MWCNT weight percentage, which improves sensing range and resolution. Such sensors can be useful for measuring high pressures in many industrial (e.g., manipulator feedback), automotive (e.g., damping elements, bushings), and underwater (e.g., depth sensors) applications.

## 1. Introduction

Pressure fields and variations are ubiquitous and provide a reliable way to measure both the state of an environment and its dynamics, with examples including weather prediction [1] and seismic activity monitoring [2]. Therefore, robust, scalable, and cost-effective pressure sensors are particularly important for observing and understanding spatially- and temporally-widespread phenomena in an environment, as well as increasing integration with an environment in contexts such as electronic skins [3] and human–machine interfaces [4]. To fully capture the information that pressure fields provide, it is critical that a wide range of pressures, and not just sub-atmospheric variations, are measurable. Here, existing methods to monitor high pressure environments are reviewed and the motivation and approach to address current challenges are described.

### 1.1. Motivation

Current polymer-based sensors typically detect low pressures (<100 kPa) with high sensitivity, but tend to saturate quickly and have a maximum pressure detection limit on the order of 10 s of kPa [5,6,7,8]. Because these sensors are optimized for low pressures, using them in high pressure environments leads to reduced measurement capabilities and sensitivity, as well as potential material failure.

Currently, high pressure sensors typically use rigid, non-compliant materials [9,10,11] that are prone to damage (plastic deformation, cracking). Sensors designed for high pressure applications also show significant measurement degradation in low pressure environments [12,13]. Therefore, a low cost, sensitive, robust, and easily integrated sensor is needed to measure the high pressures found in multiple applications, including industrial (e.g., manipulator feedback), automotive (e.g., damping elements, bushings), and underwater (e.g., depth sensors) monitoring.

For high pressure composite sensors, elastomers are desirable matrix materials due to their elasticity (i.e., reversibility), low cost, and ease of fabrication. Elastomers are also incompressible and can withstand high pressures without material failure. However, the most commonly used elastomers have a low Young’s modulus, limiting their effective pressure sensing range.

To measure pressures in the range of 1–100 atm (approximately 0.1–10 MPa), an elastomer with a high Young’s modulus is needed to avoid complete compression within the pressure range, while sensors implementing Polydimethylsiloxane (PDMS) as the matrix have had success with implenting sensors over a wide range of pressures [14,15,16,17], and the pressure range of interest in this work far exceeds what PDMS is capable of. The maximum Young’s modulus of PDMS (as a function of the monomer to curing agent ratio) has been shown to be 2–3 MPa [18,19]. This range has been significantly extended in some cases (up to 10 MPa [20]) but is still insufficient for the pressure range of interest.

Compared to the low modulus silicone-based elastomers used in low-pressure composite-based sensors, thermoplastic polyurethanes (TPUs) can increase the pressure range of elastomer-based composite sensors due to their high and tunable modulus. The modulus is controlled by the ratio of hard- and soft-segment molecules used in the TPU formulation.

### 1.2. State of the Art

Polymer-based sensors have been explored extensively [8,21,22,23,24], as have TPU-MWCNT (multiwalled carbon nanotube) composite sensors specifically [6,25,26,27,28,29]. Several groups have also researched the imposition of a nano-scale structure into devices made with such composite systems. Li et al [8] employed sacrificial cast-etching to synthesize a nanoporous sensing material able to measure pressures down to single-digit pascals. Huang et al. [28] leveraged freeze-casting to implement anisotropic microfeature templating, enabling similar sensitivity, as well as directional pressure sensing due to the orientation of the microstructures in the material. Rizvi et al. [29] introduced small scale features into a TPU-CNT composite by utilizing a pressure and temperature quenching-based material synthesis.

Although these approaches successfully developed cost-effective and sensitive devices, the primary focus was to improve the low-pressure (10 s of kPa) sensitivity. This is typically achieved by increasing CNT loading (measured in wt%) and/or introducing microscale features to increase the interfacial surface area [30,31,32]. This increased low-pressure sensitivity comes at the cost of high-pressure sensing and sensitivity, which leads to the saturation of the response at relatively low (<<1 atm) pressures.

### 1.3. Approach

This work investigates high pressure, low cost, polymer-CNT composite pressure sensors. With reusability and longevity in mind, limiting the maximum sensor strain experienced at the target conditions is of interest in order to prevent matrix failure, promoting sensor robustness overall. A modulus of at least 20 MPa was used as a lower threshold in this work to limit the maximum strain percentage to 50% at 10 MPa applied pressure.

First, a composite material to form the sensor is synthesized by dispersing MWCNTs, of sufficient concentration, in a solvent to establish homogeneous conductivity throughout the composite. Sufficient MWCNT dispersion during synthesis allows the percolation threshold and sensor response to better align with the the properties of the nanotubes, rather than their agglomerates (resulting in lower conductivity). This is important because the percolation threshold is both concentration and geometry dependent and the agglomeration of MWCNTs results in a different apparent size and geometry than that of the constituent MWCNTs (e.g., large spheres versus long, thin wires). TPU is then added to the solvent and MWNCT solution, which is thoroughly mixed to enforce homogeneous MWCNT dispersion throughout the solution. The sensors are then formed from the resulting composite material using injection molding and are characterized with a dynamic testing system. The relevant testing data (force, displacement, resistance, timestamp) are collected for offline processing and analysis. A design of experiments (DoE) is conducted, and statistical analysis is used to characterize the influence of several composite synthesis parameters (MWCNT loading, solvent type, TPU moduli) on the observed sensor response.

Unlike the more traditional one factor at a time (OFAT) experimental approach, a DoE identifies how both factors in isolation (main effects) and interactions between factors affect the measured response. Classical designs, such as factorial or fractional factorial designs, require the boundaries of the experiment to fit specific parameters leading to limited flexibility, a strict number of experiments, and restrictions on the factor types and number of levels. Optimal designs afford greater flexibility by creating an experimental design according to the boundaries of the problem at hand and allow for mixed factor types with any number of levels. As this study involved both categorical and continuous factors with a mixed number of levels, an I-optimal DoE was used, which aims to increase the precision of the response prediction for a given treatment [33].

A *p*-value for multi-factor analysis is calculated to determine if an effect is significant. The test statistic of ANOVA is the F-statistic (mean square treatments (MST)/mean square error (MSE)), which compares the variability between treatments to the variability within a given treatment (the variability of the population vs. the variability between replicated samples that should be identical). Iterative model analysis can reject non-important factors and characterize the effects of the significant factors found. This is particularly powerful because, after non-important factors have been identified, the analysis can be modified so that they are not examined, allowing existing data to be used to increase the power of subsequent designs.

## 2. Methods and Materials

A TPU-MWCNT composite material is synthesized to form the sensors studied in this work. As agglomeration can affect the homogeneity and response of the composite material, solution-based processing (as opposed to the more typical melt-mixing processes) is used to prepare the composite. The synthesized material is injection molded to form the material into the final sensor geometry. The sensor is then cyclically compressed and the relevant response data are collected and processed. The processed data from the optimal design of experiments are then analyzed to determine the effects of tracked synthesis parameters (and their interactions) on the sensor response.

### 2.1. Percolation Threshold and Process Parameter Selection

The percolation threshold is the concentration of a filler material in a matrix that maximizes the probability of long-range connectivity between fillers. The percolation threshold is estimated by Equation (1) where σ is the conductivity of the composite material, $\sigma_{0}$ is the filler conductivity, Φ is the volume fraction of the filler in the composite, and $\Phi_{C}$ is the critical value of the filler volume fraction (percolation threshold). The exponent *t* is a fitting parameter based on the dimensionality of the filler, and has been shown both empirically and theoretically to be between one and four for polymer-carbon nanoparticle composites [34]:

(1) $$\begin{array}{c} \Phi_{C}=\Phi -\sqrt[{t}]{\frac{\sigma }{\sigma_{0}}} \end{array}$$

If the MWCNTs are not homogeneously distributed throughout the precursor solution, agglomeration of the MWCNTs can occur, forming clumps and changing the apparent sizes and electrical properties of the additive [10,35]. Such agglomeration can affect both Φ and *t*, changing the percolation threshold drastically. Changes to the percolation threshold of a phase in a composite directly impact the amount of the phase necessary to establish long-range connectivity (and thus conductivity and sensitivity in this case) throughout the sample. The processing parameters for composite synthesis (outlined in Section 2.3) were selected to maximize the dispersion of MWCNTs in dissolved TPU. Multiple mixing steps are expected to improve the composite uniformity and lead to greater sensor reliability. In addition, the effects caused by variations in processing parameters (i.e., mixing time, additive dispersion, etc.) are minimized, providing a more reliable characterization of the resulting samples. Furthermore, establishing a standardized synthesis procedure minimizes variations in the processing parameters, allowing for direct comparison of multiple samples.

### 2.2. Design of Experiments

Following the composite material synthesis in Section 2.3, 34 total sensors were fabricated with varying combinations of factor levels (shown in Table 1 and Table 2). The sensor treatments were determined using an optimal design of experiments that was generated by JMP [36] to estimate all main effects, second-order interactions, and quadratic terms. The measured response data were then cleaned (outliers removed via thresholding) and linear regression via standard least squares was used to fit a model to the remaining data.

An optimal DoE was used to characterize how three factors (TPU formulation, solvent type, and MWCNT weight percentage) affected the span of the normalized response of the implemented sensors. The span of the response range was chosen as the output factor of interest because the sensor response typically approaches the end of its range asymptotically, and so sensors that have a wider response range will be able to better resolve a given pressure range. The span of the response was normalized to be able to compare sensors whose response might not be in the same order of magnitude (e.g., kΩ–GΩ). Because there are several composite synthesis aspects, some of which may even interact each other to affect the response of the sensor, an efficient method for examining the design space is beneficial. While examining each aspect (input) in isolation would provide insight into their effects on sensor response, little about their potential interactions can be learned in this way. Additionally, the number of samples needed for such a study would be large (≥60 for the number of factors explored). Because of these considerations, a design of experiments (DoE) was implemented to examine the response behavior and dependencies of the sensor.

### 2.3. Material Synthesis

**Figure 1:**
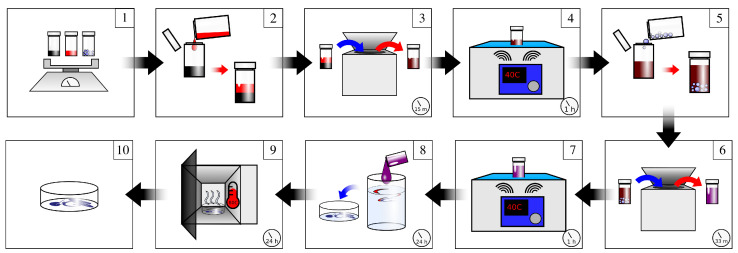
Schematic of synthesis process. MWCNT (black), TPU (blue), and solvent (red) amounts are calculated and measured (**1**), and the solvent is added to the MWCNTs (**2**). The mixture (red and black) is then stirred (**3**) and ultrasonicated ((**4**), brown). TPU is then added (**5**), and the solution (brown and blue) is stirred ((**6**), purple) and ultrasonicated (**7**) again. The mixture (solvent, MWCNTs, TPU) is then rinsed in water ((**8**), dark blue) and dried (**9**). After drying, the material is attained (**10**) and is ready to be injection molded.

The composite material used to fabricate the high pressure sensors is synthesized using several mixing steps with parameters chosen to maximize the homogeneity of the precursor solution, minimize MWCNT agglomeration, and decrease the percolation threshold. The synthesis procedure is outlined in Section Procedure, and is presented schematically in Figure 1. A revolutionary mixer (Kurabo Mazerustar KK-250s) was used in steps three and six. An ultrasonic bath (SharperTek XPS120-3L) was used in steps four and seven, and all constituent materials were weighed in a fume hood. A vacuum oven (Thermo Scientific 3608, Waltham, MA, USA) was used to dry the composite material after mixing steps. All samples (34 total) were made using the same procedure and conditions, outlined in Section 2.2. The TPUs used were polyether-type formulations with different Shore Hardness (Estane 58881, Estane 58219, Lubrizol Corp, Wickliffe, OH, USA, 80A and 92A, respectively). MWCNTs with an outer diameter of 10–30 nm and a length of 10–30 μm (US4500, US Research Nanomaterials, Inc., Houston, TX, USA) were used. The MWCNTs were agitated in one of three different solvents to ensure dispersion prior to adding the TPU to be dissolved. The solvents used were N, N dimethylformamide (DMF, Sigma Aldrich, St. Louis, MA, USA, DX1730-1), tetrahydrofuran (THF, Sigma Aldrich, 401757-2), and THF and Acetone (Sigma Aldrich, 179124-2.5L) in a 4:1 (80%/20%) mixture (THF-A).

#### Procedure

1. Calculate the mass of MWCNTs, based on the loading level (in a given volume of TPU). Weigh/measure TPU, MWCNTs, solvents and set aside;
2. Add solvent (DMF, THF, THF-A; 20 mL) to MWCNTs (solutions processed in 20 mL glass vials);
3. Mix solvent and MWCNTs (setting: 3–9; 880 revolutions per minute, 1.0 rotation/revolution ratio, 990 s);
4. Place in ultrasonic bath (40 °C, 1 h);
5. Add TPU;
6. Mix (setting: 3–9, 1980 s);
7. Place in ultrasonic bath (40 °C, 1 h);
8. Add solution to stirring water (≥250 mL). This drives off and dilutes the solvent, allowing the MWCNT-laden TPU to solidify. After mixing for ≥5 min, larger pieces of material are cut to a size of ≤20 mm on a side. This provides greater surface area for continued removal of the solvent, while still remaining large enough to be handled and not so small that pieces become airborne in later steps. Then, replace water and mix (STP, 24 h). This ensures that solvent is driven off from the synthesized material as much as possible.
9. Remove material from water and dry in a vacuum oven (≥20 in Hg, 100 °C, 24 h);
10. Final material is attained and is ready for injection molding.

### 2.4. Sensor Fabrication

**Figure 2:**
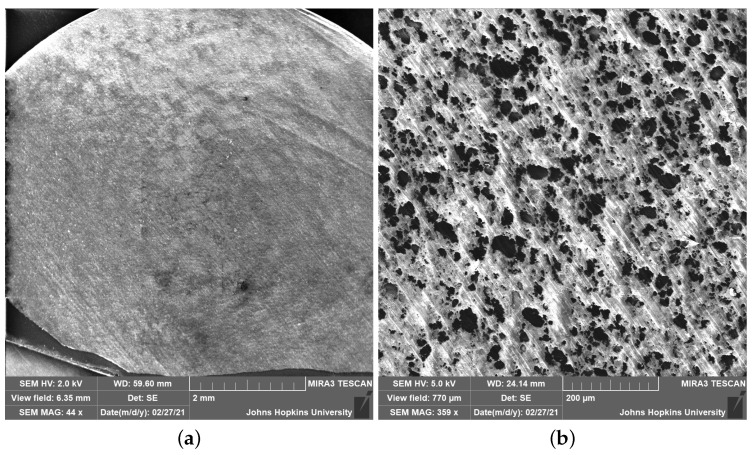
Representative SEM image of sensor cross section to examine internal morphology. SEM imaging was used to characterize internal morphology of formed sensors and determine the efficacy of the synthesis and fabrication procedures. (**a**) No voids or pitting demonstrate efficacy of injection molding, while minimal charging on non-coated composite material demonstrates conductivity. Absence of charged regions (at the scale of the entire cross section) demonstrates uniform distribution of MWCNTs. (**b**) Zooming in allows more charges to accumulate on non-MWCNT-laden regions, causing them to charge up and swell—both of which provide increased contrast to visualize the presence of MWCNTs and verify dispersion uniformity.

Once synthesized and dried, the composite material must be formed into a known geometry to reliably characterize and compare sensing performance. Injection molding was used to form the sensors because of its fast fabrication time (approximately 20 min to form a sample, including heating and cool down time) and ability to make many samples with identical geometries and internal structures. The process forms uniform samples by heating the composite material and extruding it into a mold. As pressure is applied, the composite material completely fills the mold and any trapped air is compressed and forced from the high pressure mold interior to the lower pressure (STP) exterior through venting ports, forming a bulk (solid) sensor (see Figure 2).

This venting also mitigates dieseling (diesel effect—the compression-driven heating/combustion of a gas) associated with trapped gasses, preventing scorching, burning, or decomposition of the composite material.

**Figure 3:**
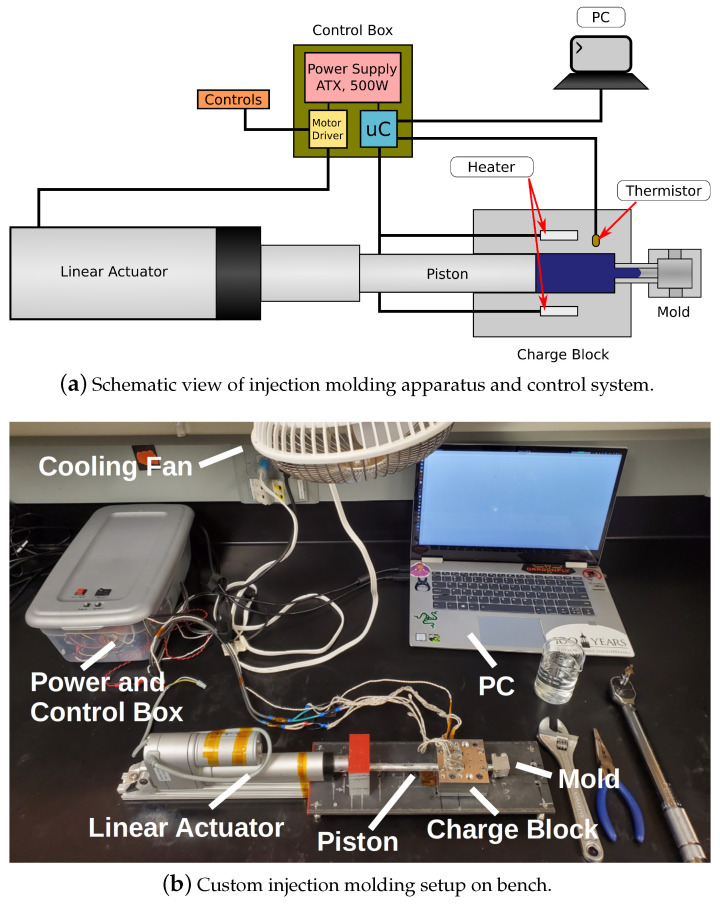
The complete injection molding apparatus. The motor driver and microcontroller (uC) are powered by the power supply. The microcontroller activates a relay to power the heaters, and the temperature of the charge block is monitored with a thermistor. The microcontroller then sends the data to a connected PC for monitoring and logging. The motor driver is controlled with an enable switch and direction buttons to drive the linear actuator forward and in reverse.

To investigate the effects of the composite material synthesis on the sensor performance, cylindrical sensors with a 10 mm diameter and height (established empirically) were formed. As relatively small volumes of the composite material are needed to form these sensors, commercially available injection molding systems are not suitable because they require a large amount of material (typically > 15 mL) for processing. To address this issue, a custom injection molding apparatus (IMA), shown in Figure 3b, was designed and built to operate with small material volumes (1.5 mL).

The IMA is shown schematically in Figure 3a. It uses four hobbyist 3D printer heater cartridges (12 V, 50 W) powered by an ATX power supply (Dell NPS-305AB A, 305 W) to heat the charge block. To control the temperature of the charge block, one thermistor (NTC 100k) is used for feedback.

To use the IMA, the mold and charge block are disassembled (top half removed from bottom half), cleaned, and reassembled with all fasteners torqued to 50 in-lbs with a 1/4 in torque wrench. The mold is attached to the charge block via a threaded coupler. The composite material is added and the piston, loosely held in place by the linear actuator (LA), is positioned in the charge block. The power supply is activated, and the temperature is tracked via a connected computer. When the temperature reaches the target temperature (190 °C), push buttons are used to drive the LA forwards, pushing the piston and injecting the melted composite material into the mold. The power supply is deactivated, with the LA maintaining pressure on the piston and the entire assembly being allowed to cool. When cooling, the sample is easily retrieved from the mold by separating the halves of the mold.

**Figure 4:**
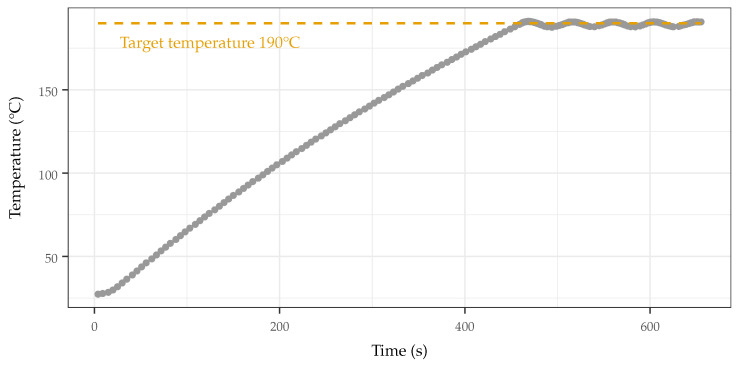
Heating profile of injection molder. Rise time is less than 8 min from cold start. Hysteresis is set to 1 °C, providing tight oscillation about the set point.

As the charge block heats up (≈8 min to the the target 190 °C) and is exposed on all sides, thermal runaway is not a concern and removing power causes the temperature of the block to stop rising quickly (≤5 s). Additionally, the on time quality of the heater elements is varied based on the difference between the measured temperature and the setpoint. This allows the temperature to increase quickly upon a cold start, as well as allow an aggressive response to sudden cooling during operation, such as during the addition of material to the charge block. The cyclic behavior of the fast increase and decrease in temperature in response to the control of power to the heaters causes a controlled oscillation about the target temperature, as can be seen in Figure 4 at 460 s. This serves to mitigate excessive oscillations and overshoot. Because of this stable and predictable behavior, a bang-bang controller was sufficient and was implemented on a microcontroller (ATtiny 84).

**Figure 5:**
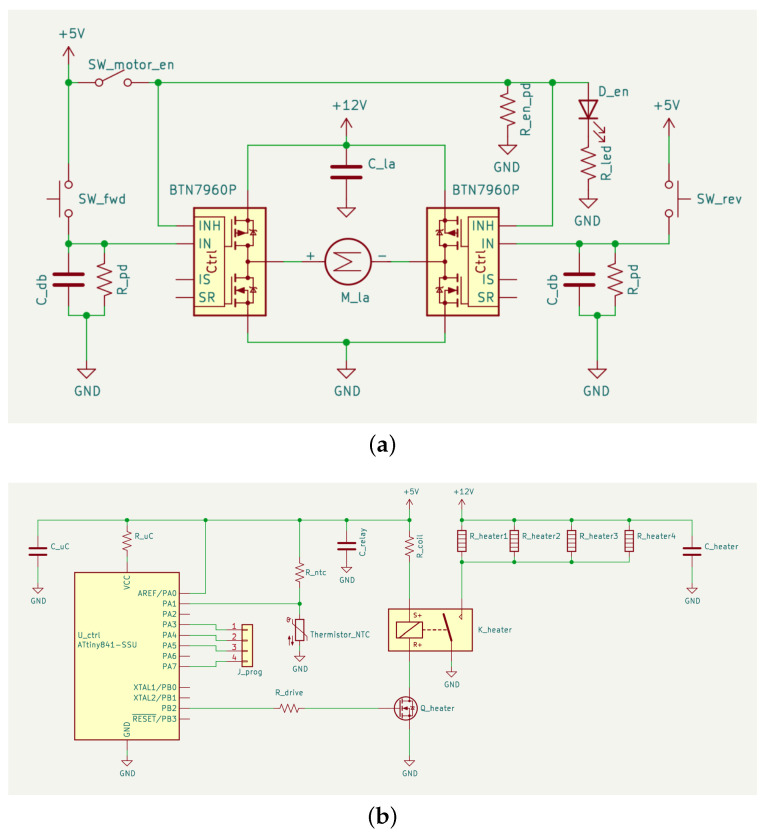
Schematics of the control circuitry of the injection molding apparatus (IMA). (**a**) Schematic of linear actuator drive circuit. The H-bridge configuration enables the linear actuator (LA) to be driven forward and in reverse. Capacitors on inputs provide debouncing, preventing spurious turn-on. Capacitors on the power rails provide an energy buffer to prevent sudden power draws from resetting the microcontroller of the heater controller; (**b**) schematic of heater controller circuit. Heaters are activated via a relay, whose control coil is activated by a microcontroller (μC) driven MOSFET, which itself has a built-in recovery diode. This isolates the μC from potentially damaging inductive spikes associated with driving the control coil.

The linear actuator (Concentric LACT4P) used to eject material from the charge block is powered from the same power supply by way of two half bridges (Infineon BTN7960P) that are controlled with two push buttons (circuit shown in Figure 5a). As the material is pushed out of the heated chamber and into the mold, any trapped air is compressed and forced out of the mold and the molded sensor, via deep landing vents located on either side of the mold cavity.

Pressure applied to the material during compression is not tracked, but the linear actuator used to apply pressure to the piston is driven to stall (DTS) during actuation, ensuring that all samples experience the maximum force that the LA is able to impart. After the initial DTS, the IMA is left to sit (heaters powered) for three minutes. The linear actuator is then DTS a second time, and left to sit powered for another 3 min. The linear actuator is then DTS a third time, the heaters are powered down, and the cooling fan is turned on, allowing the molded sensors to cool and solidify before being removed from the mold. After being removed from the mold, the formed sensor is left to sit for 24 h before testing to allow for complete cooling and relaxation of residual stresses from the injection molding process.

### 2.5. Measurement and Data Collection

Sensors made with composite materials typically experience a permanent change in resistance during the first few (≤15) cycles [23,32,37], hereafter referred to as “break in”. As such, the fabricated sensors are cyclically exercised before characterizing their performance.

During this break-in period, for each cycle, a sample is preloaded with 5 N of compressive force. After a 60 s delay, the sample is compressed at a rate of 50 N/s to a final loading of 800 N. These break-in conditions are chosen because they simulate the conditions the sensors experience after multiple cycles of the characterization cycle. Furthermore, the accelerated loading of the break-in cycles exposes the sensors to greater stress and strain rates than are experienced during the characterization cycle, ensuring that any changes to the sensor or response properties occur before characterization. The sample is then unloaded at a rate of 90 N/s to the preload force, and the cycle repeats. During the characterization cycles (first and last cycles of the routine), the sample is compressed at a rate of 5 N/s to a final loading of 800 N.

**Figure 6:**
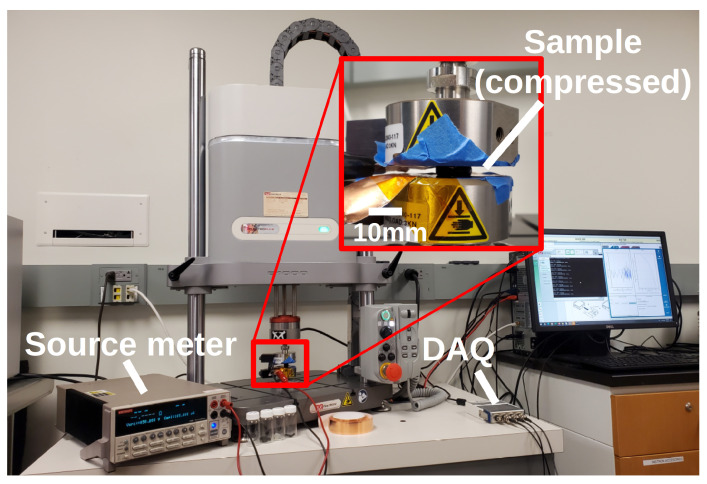
Characterization and data collection setup. The DAQ is used to collect deformation information from the dynamic test system while measurements are read simultaneously from source meter as a sensor is compressed. Pieces of copper tape on either face (that are isolated from the frame of the test system) are used as electrodes.

Samples were compressed using a dynamic test system (Instron E1000, “Instron” hereafter). Applied force and subsequent displacement were reported by the Instron and converted to analog voltages onboard. These voltages were measured with a data acquisition unit (DAQ, NI 9234 and NI USB-9162 carrier) and the sensor resistance was measured with a source meter (Keithley 2400) configured to provide 2 V with a compliance current (maximum current allowed in attempting to establish the requested voltage) of 105 μA. Copper tape was used to make electrical connections with the sample under test, and an insulating tape prevented electrical connections between the sample and the grounded Instron frame. The complete testing setup is shown in Figure 6.

### 2.6. Characterization

The response of similar composite-based sensors is typically modeled via a piecewise linearization with two or three sections [8,30]. These piece-wise approximations do not fully capture the curvature in the sensor’s response. This is typically not an issue for low pressure sensors, as the missed information corresponds to 10 s–100 s of pascals. For the high pressure sensor presented, however, such omissions would lead to gaps or inaccuracies on the the order of 100 s of kPa (≈1 atm). To capture as much information as possible, the sensor response (resistance vs. applied pressure) was characterized as a biexponential function (Equation (2)), which approximates the typical response shape observed in both the measured samples and existing literature:

(2) $$\begin{array}{c} y\left(x\right)=A_{1}e^{k_{1}x}+A_{2}e^{k_{2}x}+c \end{array}$$

Prior to biexponential fitting, the collected raw data were separated by cycle, cleaned, smoothed, and filtered. Cleaning entailed removing erroneous measurements (negative resistances), exceptionally high resistances (≥1 TΩ), and cycle markers in the data, and rounding calculated values to three decimal places. Smoothing was implemented by convolution with a Gaussian kernel with a width of one (chosen empirically). Filtering was performed by first fitting a biexponential curve to the response of a given sample (for each cycle individually).

The fit was then used to predict values of the response for a given pressure, and the standard deviation of the error and the root mean square error (RMSE) were calculated. If the error associated with a data point with respect to the fit of the data is more than two standard deviations from the RMSE, it is considered an outlier and is removed. After doing so, a new biexponential is fit to the remaining data. The outlier removal improved the fit by removing data points that skewed the biexponential parameters. The outlying measurement points were likely due to very high or very low readings reported erroneously from the source meter or caused by noise on the sensing lines. After this process is applied to all cycles for all samples, the final cycle for each sample is used to characterize its response. The final cycle was used because any permanent changes to the sample response (break-in) occur in the first few cycles after fabrication. The number of break-in cycles ranges from 5 to 15 in literature, and so greater than 20 cycles were used. It was observed empirically that, after 20 cycles, the sensors exhibited similar behaviors among subsequent cycles (shape, response range).

**Figure 7:**
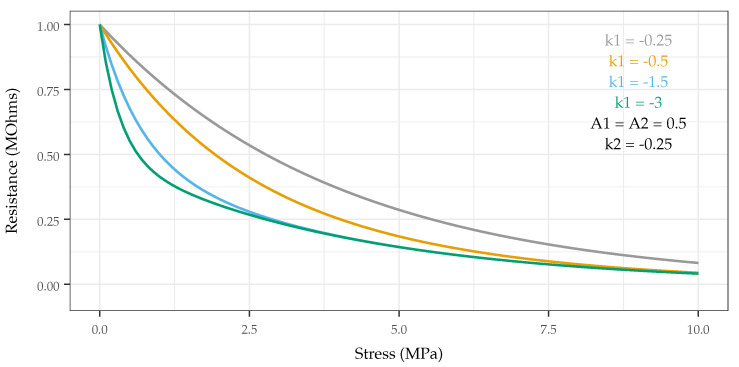
Illustration in the change in shape of the biexponential function as a function of exponential coefficients. With one coefficient fixed, the second is varied from −0.25 to −3, demonstrating the change in shape and affected region of the curve.

The original output parameters chosen for examination were the coefficients in the exponents of the function (k1 and k2, respectively), as they determine shape of response (see Figure 7). However, these parameters were dropped from consideration because k2 and the ratio of k1 to k2 showed little correlation with the chosen treatment (input) parameters (significance level of *p* > 0.05 used). K1 demonstrated showed statistically significant correlation with MWCNT loading (discussed in Section 3.1) but only characterizes the lower pressure regime of the sensor. The normalized span of the measured resistances (with respect to the initial value for a given sensor) was chosen because (1) increasing span effectively increases resolution and sensed pressure separation, and (2) comparing values of the same order of magnitude provides a better basis of comparison for sensors whose responses span several orders of magnitude (kΩ to GΩ) depending on treatment.

## 3. Results

Foremost, it was demonstrated that, through careful selection of the matrix and its properties, as well as processing parameters, the pressure sensing range of the composite sensor can be controlled and significantly extended. The sensor is able to detect pressures up to at least 10 MPa (limits of the pressure testing setup) versus the 10 s of kPa for polymer composite pressure sensors reported in literature, as shown in Table 3. It was also found that a decreased carbon nanotube loading was correlated to an increase in response sensitivity at high (≥3 MPa) pressures.

**Figure 8:**
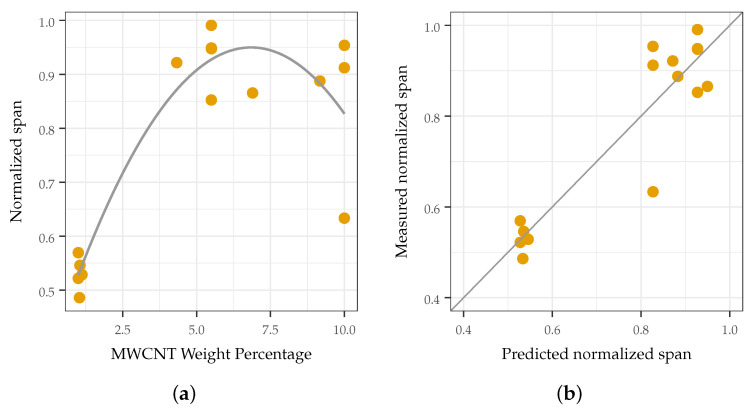
The normalized span with respect to CNT loading and the goodness of fit of the prediction of the span. (**a**) normalized span of sensing response with respect to MWCNT loading. The effect of TPU stiffness was not found to be significant (p>0.05); (**b**) prediction of normalized span.

**Figure 9:**
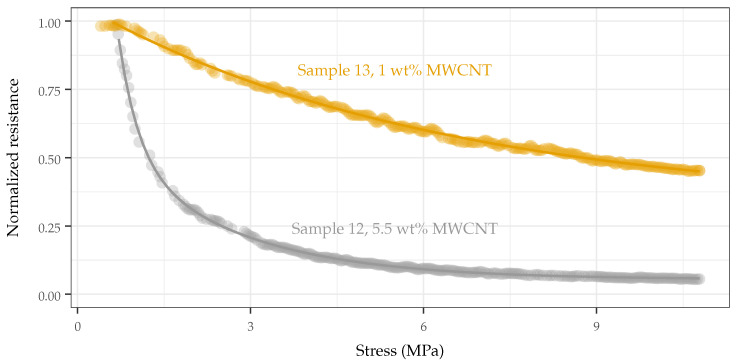
Response (normalized) of samples made with identical TPU formulations (Estane 58881, Y = 23 MPa) and solvent (THF), but varying MWCNT loadings (Sample 12: 5.5 wt%, Sample 13: 1 wt%). As the CNT loading decreases, the span of response increases (corresponding of an increase from 18 MΩ to 8 GΩ), providing better differentiation/resolution for a given pressure.

It was found that increasing the MWCNT loading did not necessarily increase the sensitivity. This appears counter-intuitive at first, as increasing the MWCNT loading improves the sensitivity for small pressures and is the defacto method for eliciting increased sensitivity from similar composites. Using this approach, similar composites have been reported to sense pressures in the 1–10 Pa range. However, this causes the response of such sensors to saturate at a lower pressure, degrading the response at elevated pressures and decreasing the effective sensing range. MWCNT loadings that were too high (such that the response of the sensor saturated quickly) or too low (such that the sensor was not responsive) led to a decreased response span, degrading sensor performance at elevated pressures, as shown in Figure 8a and Figure 9. The predicted response span matched well with those measured (see Figure 8b), demonstrating a causative correlation between the synthesis parameter and the response variable. The analysis and verification of these findings are discussed in Section 3.1.

**Figure 10:**
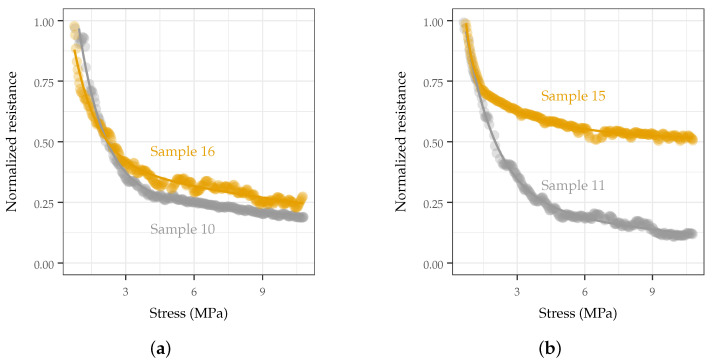
Representative samples of behaviors seen with different TPU formulations. The higher modulus formulation demonstrated poor repeatability among replicates, indicating non-reliable dispersion of the MWCNT additive in the composite material with the given processing steps and parameters outline in Section 2.3. (**a**) Softer TPU (Estane 58881) replicates demonstrated similar response shape and behavior span; (**b**) example of stiffer TPU (Estane 58219) replicates demonstrating significantly different behaviors.

TPU formulation affected the response range of the sensors, as most of the formulations using the stiffer TPU formulation needed to be removed due to lack of response and non-repeatable measurements (see Figure 10). The effect of TPU stiffness did not correlate with any of the tracked parameters, and potential causes and remedies are discussed in Section 4.

**Figure 11:**
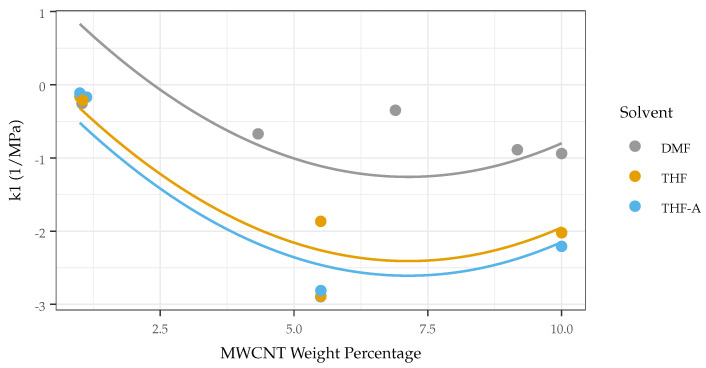
Change in k1 (pressure onset sensitivity) as a function of MWCNT loading and solvent type, starting near zero when MWCNT loading is low, as expected. It is then shown to increase (in magnitude) as MWCNT loading increases. This sensitivity then starts to decrease as MWCNT passes the point of maximal sensitivity, as the sample becomes so conductive that pressure no longer significantly actuates the sample resistance.

While k1 was shown to have a relationship with MWCNT loading (see Figure 11) in accordance with the literature, it was found that k2, as well as the ratio of k1 and k2 (the overall shape of the response), did not demonstrate significant correlation (*p* > 0.05) with the explored treatment parameters. However, the shape of the response did exhibit different behavior across treatments, implying that varying the carbon nanotube loading, matrix type, or solvent has little effect on the shape (relative sensitivities in different areas of the response curve) of the response. K1 is shown to increase towards maximum sensitivity (as a function of MWCNT loading) at a pressure that aligns with the maximum span as a function of MWCNT loading. This reinforces that span and sensitivity (at least at the lower end of the pressure range) are related and that the behavior of the sensor is in accordance with findings and mechanisms found in existing works.

### 3.1. DoE Analysis

After cleaning the data as outlined in Section 2.6, the data were checked to confirm a good fit between the measured data and the generated fit. The treatment used to fabricate each sensor (see Table 2) was referenced and replicate treatments that did not produce matching curves were removed (see Figure 10). The DoE was then analyzed iteratively. First, samples that were not conductive were removed. For the remaining samples, the root mean square error (RMSE) was divided by the span of the normalized response to provide a metric that quantified how closely the predicted and measured data matched throughout the measurement range (hereforth referred to as the “metric” of a sample). This metric was chosen to quantify how well the biexponential curve fit the measured data, mitigating the effect of heavy tails skewing the metric. This was necessary as the curve that was fit to a response did not necessarily overlap with the response data throughout the response range.

The metrics of all samples were plotted on a histogram, and samples with metric in or above the 85th quantile were removed due to significant deviation between the fitted biexponential and the measured data. Analysis was then performed iteratively on the remaining data to maintain statistical power for examined factors. Primary effects were examined in isolation, and non-significant effects were dropped from consideration. Secondary effects containing significant primary effects were then considered. Following this approach, few (5 of 16) samples made from treatments that comprised the comparatively stiffer TPU formulation remained. Of those that remained, treatments with similar parameters had significantly different responses (discussed in Section 4) and so all remaining samples made with this TPU were removed. The effects of MWCNT weight percentage and solvent on the normalized span of the remaining TPU samples were assessed via standard least squares linear regression. Factors with a *p*-value greater than the significance level of 0.05 were eliminated via backwards removal.

**Figure 12:**
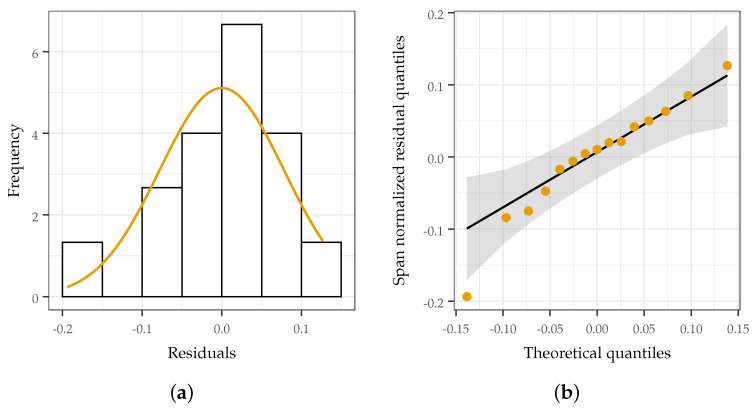
Examination of residual distribution. A normal distribution with a mean of zero implies that error is random and not a result of treatment or untracked parameters. (**a**) histogram of residuals. Bars match overlaid normal distribution well, indicating that a zero-centered normal distribution is a good fit; (**b**) QQ plot (residuals vs quantile) of experimental data. Residuals are approximately linear in this space with points being grouped in the middle quantiles and spreading out in higher and lower quantiles, indicating that larger-magnitude residuals are fairly rare.

**Figure 13:**
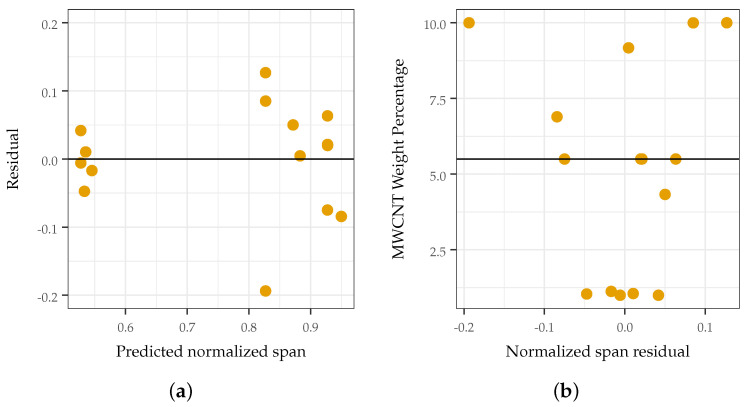
Examination of error correlation. No observable pattern in either plot implies that the observed error is random. (**a**) Residuals do not exhibit any pattern with respect to the predicted response range (normalized resistance), indicating no correlation. (**b**) MWCNT vs. residuals do not demonstrate any pattern, indicating that error is not a function of loading treatment.

In order to validate the analysis, the error and its distribution were examined. In analyzing the error, a histogram (Figure 12a) and QQ plot (Figure 12b) of the error were generated to visually confirm that the error was normally distributed with a mean of zero. A Shapiro–Wilk test was also conducted (*p* = 0.53) to ensure good overlap between the distribution of the error and a zero-centered normal distribution. Plots relating the residuals to the predicted span (Figure 13a), MWCNT loading (Figure 13b), and treatment ordering exhibit no discernible pattern, demonstrating constant variance and normal distribution.

### 3.2. Hydrostatic Pressure Sensing

A use case for the presented sensor is pressure measurement in hydrostatic environments, such as those encountered in the ocean. In these extreme-pressure environments, conductivity, temperature, and depth (CTD) sensors are typically employed for environmental measurements. The presented sensor can serve as a low-cost solution for implementing the depth sensing functionality in such a system.

**Figure 14:**
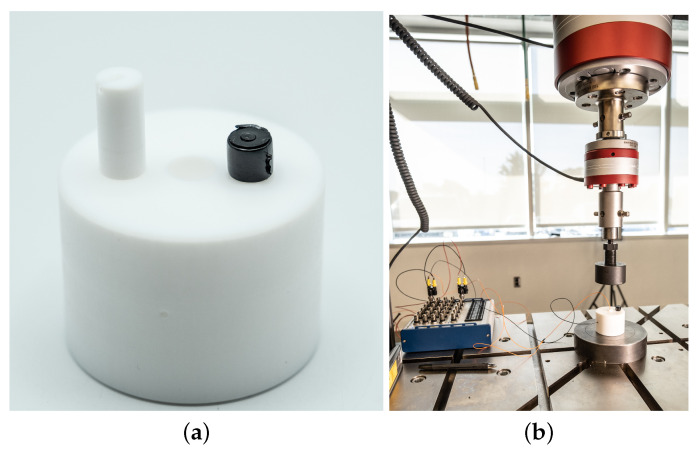
The testing setup and apparatus for simulating hydrostatic loading. The rigid die prevents the sample under test from expanding perpendicular to the loading direction (via reactionary forces), applying a similar force to all sides of the test sample simultaneously, simulating a hydrostatic environment. (**a**) Close up of Teflon piston and die, as well as one of the piezoresistive nanocomposite-based sensors used in testing; (**b**) testing setup for simulating hydrostatic loading.

The hydrostatic loading experienced will cause a different sensor response curve with respect to the axial compression presented thus far; however, sensing range is still a function of MWCNT loading. Figure 14a shows the compression die (made of Teflon) for the testing of the fabricated sensors. The sample is placed in the die, and pressure is applied to the sensor via the piston shown using a dynamic testing system (see Figure 14b).

**Figure 15:**
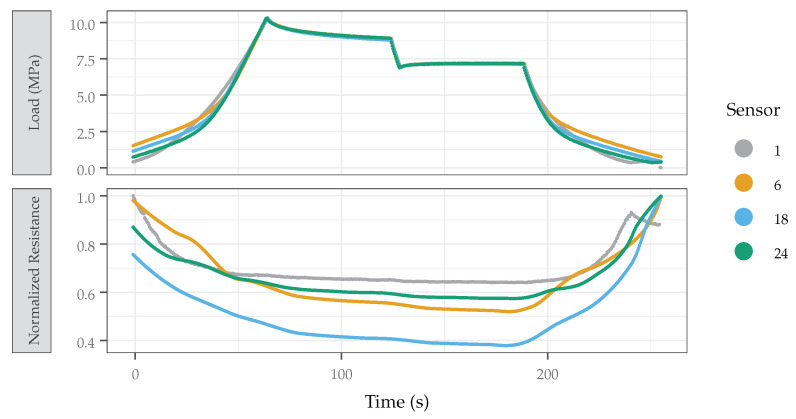
Sensor response (normalized) and applied pressure plotted with respect to time. The response of the sensor at the timescale of interest was observed to be effectively instantaneous at a load rate of 0.172 MPa/s (1.7 atm/s, 17 m/s) and a sampling rate of 5 Hz (0.2 s intervals), equating to a sensor response time of, at most, 0.2 s. Sensor 18 demonstrated the highest relative resistance change and thus the best performance.

When compressed axially without a die, the samples expand perpendicular to the loading direction. The die restricts this expansion by applying pressure to the sample from all directions, simulating the isometric, hydrostatic loading experienced underwater. As shown in Figure 15, samples 1, 6, 18, and 24 are compressed in the compression die and their responses recorded. Sensor 18 demonstrated the highest resistance change, and thus the best performance. Samples 1, 6, and 24 exhibited similar behavior but demonstrated a slightly smaller change in resistance.

Pressure is also applied cyclically to measure the response time of the sensor. The responses of the sensors from cycle to cycle were consistent, with Figure 15 showing representative curves (last cycle was chosen, arbitrarily). This response time is shown to be effectively instantaneous at a load rate of 0.1723 MPa/s, equating to a dive rate of about 17 m/s. Resistance was sampled at 5 Hz, equating to a response time of at most 0.2 s. These measurements demonstrate the efficacy and plausibility of the use of this sensor as a pressure transducer in a CTD sensor suite.

## 4. Discussion

It was found that the shape of the response (relative sensitivities at different pressures) did not demonstrate significant correlation (*p* > 0.05) with the explored treatment parameters but did exhibit different behavior across treatments. This implies that the variation in the shape of the response is caused by material properties of the solutions of different treatments that were not varied or controlled for, such as viscosity, mix time, or mix speed. While the exponential coefficients of the biexponential fits of the sensor responses did not exhibit good correlation, a trend was still apparent. Because of this, the normalized span of the response was examined, as it is affected in part by the sensitivity of the sensor, as well as by the differences in the material and electrical properties resulting from the treatments applied. Normalization was applied to compare samples of varying orders of magnitude, as biexponential fits can vary significantly when scale changes, which is exacerbated when the response of the sensor is near the transition point of the curve defined by the fit. Normalization allowed the response of each sensor to be compared to itself, facilitating performance comparisons across sensors.

It was also found that all of the samples that used the softer TPU formulation demonstrated some response to pressure, whereas all of the samples that were found to be non-responsive used the stiffer TPU formulation as a matrix. Furthermore, the samples using the stiffer TPU as a matrix did not exhibit repeatable properties, with some repeats being dissimilar from each other. This implies that: (1) matrix choice does have a significant effect on additive dispersion and sensor performance (although it could not be quantified in this study); (2) an uncontrolled fabrication parameter has a significant effect on the resulting composite properties at the given processing parameters; and (3) the aforementioned fabrication parameter is likely related to, or a function of, the matrix choice. The most likely parameter is viscosity, which was not controlled for and would have a significant impact on how well the composite solution is mixed, and would vary based on matrix type if a fixed amount of solvent was used across treatments. Ineffective or incomplete mixing would then have an effect on sensor conductivity, likely by way of either variation of the percolation threshold as a function of matrix-related additive agglomeration, or inadequate MWCNT dispersion/composite homogeneity. This can be explored by conducting a similar DoE, which instead controls or modulates the solution viscosity and different process parameters related to MWCNT dispersion (mixing speed time, intensity, etc.), in addition to matrix type and MWCNT loading.

## 5. Conclusions

Pressure sensing is an important capability in measuring and interacting with environments. As such, being able to sense a wide range of pressures (including those ≫ 1 atm) is critical in enabling such capabilities and expanding the space of applications associated with them. By selecting a matrix material of appropriate stiffness and targeting suitable MWCNT loadings, it was possible to implement a soft composite material to be used in a sensor capable of measuring pressures in excess of 10 MPa.

MWCNT dispersion is an important factor for increasing composite homogeneity and reducing additive agglomeration. Agglomeration and dispersion affect both the apparent volume fraction of the filler in the composite (Φ) and the dimensionality-based fitting parameter (*t*), which change the percolation threshold ($\Phi_{C}$). As such, it is important for the apparent sizes of the additive particles to be as close to their true sizes as possible to minimize the effects of agglomeration and ensure that the behavior of the response is only due to the properties of the composite.

An optimal design of experiments was used to explore the relationships of composite formulation on sensor response shape and sensitivity, which allowed for the examination of the interaction of effects as well as determining which parameters had a significant effect on the examined output. In exploring the effects of processing on the shape of the resistance response, the values of the exponential coefficients were found to have a weak correlation (*p* > 0.1) with any of the tracked treatment parameters, implying that varying the carbon nanotube loading, matrix type, or solvent has little effect on the shape (relative sensitivities in different areas of the response curve) of the response.

Surprisingly, increasing MWCNT concentration was shown to decrease sensor performance at elevated pressures, which is different from the current approaches to improving the performance of similar composite-based sensors for low pressure sensing. Increased MWCNT concentration caused the response to saturate at a lower pressure, degrading the response at elevated pressures and thus decreasing the bandwidth of the sensor. Such a sensor is well suited for measuring pressures in industrial, automotive, and underwater applications due to its ease of fabrication and integration, repeatable response, good sensitivity, low cost, reusability, and overall robustness.

## Figures and Tables

**Table 1:** Factor levels used in the design of experiments. TPU formulation is varied to observe the effect of the Young’s modulus (Y) of the matrix, solvent type is varied to study any effects resulting from dispersion or solvent properties, and MWCNT weight percentage (wt%) is varied to examine any effects on conductivity and response range.

Factor	Levels
TPU formulation	Estane 58881, 58219 (Y = 23, 62.1 MPa)
Solvent type	THF; DMF; THF-A
MWCNT wt%	1–10

**Table 2:** Treatments used in the design of experiments.

Sample No.	Solvent	Y*_TPU_*	MWCNT wt%
1	DMF	62.1	10
2	DMF	62.1	1
3	THF-A	23	5.5
4	THF	23	5.5
5	THF-A	23	5.5
6	THF	23	10
7	THF	62.1	1
8	THF-A	23	1
9	THF	62.1	10
10	DMF	23	10
11	DMF	62.1	5.5
12	THF	23	5.5
13	THF	23	1
14	DMF	23	4.3
15	DMF	62.1	5.5
16	DMF	23	10
17	DMF	23	6.8
18	THF-A	23	10
19	THF-A	62.1	10
20	THF-A	62.1	1
21	THF-A	62.1	5.5
22	THF	62.1	1
23	THF-A	62.1	10
24	THF-A	23	10
25	DMF	23	1
26	THF	62.1	10
27	THF-A	62.1	10
28	THF	23	10
29	DMF	62.1	10
30	THF	23	1
31	THF-A	62.1	3
32	THF-A	23	1
33	DMF	23	7
34	THF	62.1	5.5

**Table 3:** Comparison of related works. Rizvi et al. tested samples to pressures of up to 50 MPa, but response is nearly saturated at 5 MPa (negligible change with respect to applied pressure).

Work	Material System	Max Pressure
Li et al. [8]	Nanoporous PDMS	25 kPa
Li et al. [7]	Woven conductive fibers	10 kPa
Zhong et al. [30]	Graphene PU foam	25 kPa
He et al. [5]	Piezoresitive graphene film	40 kPa
Jung et al. [16]	Au serpentine nanomembrane	50 kPa
Rajendran et al. [14]	PDMS-MWCNT film	330 kPa
Fekiri et al. [17]	DIW PDMS-MWCNT on PDMS substrate	1.132 MPa
Rizvi et al. [29]	Porous TPU-CNT	5 MPa
This work	TPU-MWCNT bulk sensor	>10 MPa

## Data Availability

Not applicable.
